# Southern Medical Reports; Consisting of General and Special Reports on the Medical Topography, Meterology, and Prevalent Diseases in the Following States: Louisiana, Alabama, Mississippi, North Carolina, South Carolina, Georgia, Florida, Arkansas, Tennessee, Texas

**Published:** 1850-07

**Authors:** 


Art. VI.—*Southern Medical Reports; consisting of General and Special Reports, on the Medical Topography, Meteorology, and Prevalent Diseases, in the following States: Louisiana, Alabama, Mississippi, North Carolina, South Carolina, Georgia, Florida, Arkansas, Tennessee, Texas. To be published annually.* Edited by E. D. Fenner, M. D., of New Orleans; member of the American Medical Association, &c., &c., &c. Vol. 1. 1849. New Orleans: B. M. Norman. New York: S. S. & Wm. Wood. 1850.

Dr. Fenner has performed a most acceptable service to the cause of medical science in the publication of this volume of Medical Reports. Its preparation could not have fallen into abler hands, as every part of the book will show to the attentive reader. These Reports are invaluable contributions to medical hydrography, topography, and statistical information.

We may convey to the reader of these remarks some idea of the character of the work, by giving the table of contents. The Reports from Louisiana are on the following subjects:

"General Report on the Medical Topography and Meteorology of New Orleans, with an account of the Prevalent Diseases during the year 1849. By the editor.

On the Inundation of New Orleans in 1816. By the Editor.

A Chapter of the Hydrography of the Mississippi river. By C. G. Forshay, A. M.

Annual Report of the New Orleans Board of Health. By E. H. Barton, M. D., Chairman of the Committee.

Special Report on the Fevers of New Orleans, Particularly the Yellow Fever of 1849. By the Editor.

Statistics of Yellow Fever and of all Diseases, in the Charity Hospital of New Orleans, for Thirty Years, from 1820 to 1850, inclusive. By J. C. Simonds, M. D.

Report on Epidemic Cholera in the city of New Orleans in 1848–'9. By the Editor.

Special Report on the Epidemic Colic, which prevailed in the city of New Orleans during the Summer of 1849. By the Editor.

Report on the Topography, Climate, and Diseases of the Parish of St. Mara, Louisiana. By James B. Dungan, M. D.

On the Cholera of Lafourch Interior. By Wm. A. Booth, M. D.

Reports on the Origin and Sanitary Condition of the Orphan Asylum of New Orleans and La Fayette.

On the Poydras Female Orphan Asylum. By J. Rhodes, M. D.

On the New Orleans Female Orphan Asylum. By O. C. Carey, M. D.

On the Male Orphan Asylum of Lafayette. By W. P. Sunderland, M. D.

On the New Orleans Charity Hospital. By the Editor.

The Louisiana State Medical Society.

Reports from Alabama.—Report on the Topography, Climate and Diseases of Madison County. By J. Y. Bassett, M. D.

Contributions to the Vital Statistics of Mobile. By George A. Cetchum, M. D.

Transactions of the Mobile Medical Society.

The Alabama State Medical Association. Report on the Prevalent Diseases of a portion of Dallas County. By F. A. Bates, M. D.

Reports from Georgia.—General Report on the Topography, Climate and Diseases of Middle Georgia. By E. M. Pendleton, M. D.

Special Report of an Extraordinary Case of Insanity. By J. C. C. Blackburn, M. D.

Medical Society of the State of Georgia.

Reports from Mississippi.—General Report on the Topography, Meteorology and Diseases of Jackson. By S. C. Farrar, M. D.

Report on Epidemic Cholera in the vicinity of Natchez. By C. H. Stone, M. D.

On Epidemic Cholera and its Preventive Treatment. By C. S. Magoun, M. D.

Disarticulation and Removal of one half of the Inferior Maxilla, for Osteo-Sarcoma. By A. M. Clemens, M. D.

On the Medical Waters of Mississippi—the Artesian Springs of Madison County.

REPORTS FROM TENNESSEE.—Report of the Commencement, Prevalence, Fatality, Treatment, &c., of Pestilential Cholera, in Memphis and its vicinity; with the prominent facts bearing upon the unsettled question of its Imported or Domestic Origin. By Lewis Shanks, M. D.

REPORTS FROM SOUTH CAROLINA.—Observations on the Fever which is developed in the city of Charleston, after exposure to the country air during the Summer and Autumn, and hence called Country Fever. By Thos. Y. Simmons, M. D.

On the Yellow Fever of Charleston in 1849. By the Editors of the Charleston Med. Journ. and Review.

Reports of Cases in Private Practice. By W. F. Holmes, M. D.

Nitrate of Silver in Anginose Affections.

Case of Paralysis treated with Strychnine.

Belladonna in Pertussis.

The South Carolina Medical Association.

REPORTS FROM TEXAS.—Report on the Topography of San Antonio, and the Epidemic Cholera that prevailed there in the Spring of 1849. By J. J. B. Wright, M. D., Surg. U. S. A.

Report of the Rise, Progress and Decline of Epidemic Cholera in the Valley of the Rio Grande. By N. S. Jarvis, Surgeon U. S. Army, stationed at Fort Brown, Texas.

EXCERPTA AND MISSELLANIES.—Remarks by the Editor.

Experimental Researches on the Action of Quinine, especially in large doses.

A Memoir submitted to the Academy of Science. By M. Brequet.

Report of M. M. Andral, Rayer, and Lallemand.

On the Treatment of the West India Remittents and Intermittents by Quinine. By Dr. D. Blair, of Demarara.

Does Calomel really expel Biliary Secreton? By Dr. Michea.

The Law relating to the Importation of Adulterated Drugs, Medicines, and Chemical Preparations into the United States.

MEDICAL COLLEGES OF THE SOUTH AND WEST.—Remarks by the Editor.

University of Louisville. Medical Department.

Medical Department of the University of Louisiana, at New Orleans.

Medical College of Georgia.

American Medical Journals.—Remarks by the Editor.

Catalogue, &c. Index."

The reader of these Reports will find in them a mass of valuable facts that cannot easily be procured elsewhere, and these facts constitute a chart that may guide the practitioner safely in his contests with disease. We hope that the physicians of the West and South will aid Dr. Fenner in his future volumes, and that each observer and writer will reflect upon the immense amount of good he may do in promoting the laudable objects of Dr. Fenner's publication. The American Medical Association, at its recent meeting in Cincinnati, resolved that "Dr. Fenner's projected annual publication on the diseases and medical statistics of the Southern parts of the United States, meets with the cordial approbation of the Association."

Dr. Fenner's book is published in very respectable style. It may be found, or ordered at the book-store of Morton & Griswold, in this city.

